# Fibroid Uterus

**Published:** 1879-11

**Authors:** Herbert W. Little

**Affiliations:** New York


PRESBYTERIAN HOSPITAL OF NEW YORK CITY—FIBROID UTERU

Miss S., æt. 48. Admitted to the hospital August 20, 1879, under the care of Dr. A. C. Post. Patient gives the following history: Mother died of phthisis; father of old age. Has three brothers and two sisters living and in good health, but has lost three sisters, one from phthisis, and two from unknown causes. None of her sisters or her mother ever had any uterine trouble.

Patient has enjoyed very good health. Has never had any sickness except her present ailment. States that four months ago began to experience difficulty in passing water; was obliged to have her water drawn for two weeks. Has not had any trouble since in passing it. Menses have always been regular until last six months; during this time she has had a leucorrhœal discharge and more or less blood with it all the time, with no regular periods. Bowels regular; no pain over uterus. Never had dysmenorrhœa. Two weeks after her trouble patient went to a physician and was told that she had a prolapsus; uterus was replaced and a pessary introduced. Caused so much pain was obliged to discontinue it.

Two weeks ago went to a different physician, who told her that she had a tumor of the uterus, which required operative interference.

Patient is a tall, spare person, with a tuberculous diathesis. Appetite good, and has not lost flesh of late. Color very good.

On examination an intra-mural tumor of the uterus was found extending down to a point just within the vulva. Presents a dark greyish appearance on the surface. There is some discharge of bloody matter, which is somewhat offensive. Ordered to be syringed twice a day with carbolic acid, 1 to 40, at a temperature of 100 deg. F., and gtt. xxx of fl. ext. ergot, (Squibb's) t.i.d. M.

On September 17th ergot had been increased to ʒj t.i.d, in hopes of bringing the tumor down by the contraction of the uterus. Tumor examined and found to be one-half inch lower, measuring four and a half inches in length.

On the 22d of September, complains of dragging pains in her back. The discharge is very offensive, being unbearable to the patient and causing depression of spirits. The tumor is projecting from the vulva for the distance of one inch, the surface of the same being dark and gangrenous, the tumor being in such a condition it was considered advisable to remove it as soon as possible.

On the 25th Dr. Post decided to remove the tumor, which he did, aided by Dr. Wm. T. White and Dr. H. W. Little, the House Surgeon, in the presence of Drs. T. G. Thomas, Jas. N. Law, C. H. Bridden, A. G. Quimby, and others. The operation was as follows, the patient first being etherized: The projecting portion of the tumor was seized with a large velsellum and drawn through the vulva, then excised with curved scissors. Different portions of the tumor within the vagina were successively seized, drawn down and excised until the tumor was reduced in bulk so that only the portion within the uterus remained. This was again seized with a velsellum and drawn down so as to put the attachment upon the stretch, when partly with the finger, but chiefly by the aid of Thomas' serrated spoon, the principal attachments were divided. The tumor was brought down through the vulva and the remaining attachments in like manner divided. The tumor being thus removed from the body, the attachments were found to be very strong, requiring considerable effort to divide them. A very moderate amount of blood was lost during the operation. The pulse, respiration and general appearance of the patient throughout the operation indicated that it was well borne. The tumor weighed 22 ounces.

Patient rallied well from the effects of the ether, and the next day was very comfortable. Some slight hemorrhage. Ordered fl. ext., ergot, (Squibbs) gtt. xv. t.i.d.

Ten days later, patient is in the hospital still enjoying better health than before the operation; appetite good; sleeps well. Nothing to complain of except a very slight sanious discharge from the uterus. The uterus is syringed with carbolic acid, one to forty, twice a day, and the ergot is still administered. The patient is still on the gain, and will be able to leave the hospital very soon.

